# Multiple Activities of LigB Potentiate Virulence of *Leptospira interrogans:* Inhibition of Alternative and Classical Pathways of Complement

**DOI:** 10.1371/journal.pone.0041566

**Published:** 2012-07-23

**Authors:** Henry A. Choy

**Affiliations:** 1 Department of Medicine, David Geffen School of Medicine at University of California Los Angeles (UCLA), Los Angeles, California, United States of America; 2 HACme Lab Services, Los Angeles, California, United States of America; University of Kentucky College of Medicine, United States of America

## Abstract

Microbial pathogens acquire the immediate imperative to avoid or counteract the formidable defense of innate immunity as soon as they overcome the initial physical barriers of the host. Many have adopted the strategy of directly disrupting the complement system through the capture of its components, using proteins on the pathogen's surface. In leptospirosis, pathogenic *Leptospira* spp. are resistant to complement-mediated killing, in contrast to the highly vulnerable non-pathogenic strains. Pathogenic *L. interrogans* uses LenA/LfhA and LcpA to respectively sequester and commandeer the function of two regulators, factor H and C4BP, which in turn bind C3b or C4b to interrupt the alternative or classical pathways of complement activation. LigB, another surface-proximal protein originally characterized as an adhesin binding multiple host proteins, has other activities suggesting its importance early in infection, including binding extracellular matrix, plasma, and cutaneous repair proteins and inhibiting hemostasis. In this study, we used a recent model of ectopic expression of LigB in the saprophyte, *L. biflexa*, to test the hypothesis that LigB also interacts with complement proteins C3b and C4b to promote the virulence of *L. interrogans*. The surface expression of LigB partially rescued the non-pathogen from killing by 5% normal human serum, showing 1.3- to 48-fold greater survival 4 to 6 d following exposure to complement than cultures of the non-expressing parental strain. Recombinant LigB7′-12 comprising the LigB-specific immunoglobulin repeats binds directly to human complement proteins, C3b and C4b, with respective K_d_s of 43±26 nM and 69±18 nM. Repeats 9 to 11, previously shown to contain the binding domain for fibronectin and fibrinogen, are also important in LigB-complement interactions, which interfere with the alternative and classical pathways measured by complement-mediated hemolysis of erythrocytes. Thus, LigB is an adaptable interface for *L. interrogans* to efficiently counteract the multiple homeostatic processes of the host.

## Introduction

Dynamic interplay between the survival mechanisms of pathogen and host determines the outcome of an infection. Pathogenic microbes have many means to enter hosts, colonize tissue, and further transmit disease. Hosts in turn have powerful cellular and humoral defense mechanisms that can repel or mitigate infections. The zoonotic disease, leptospirosis, is transmitted from reservoir hosts, such as the rat, via the exposure of accidental hosts, such as humans, livestock, and dogs, to freshwater and soil contaminated with *Leptospira* spirochetes shed in rodent urine [1], [2], [3], [4], [5], [6], [7], [8]. The common routes of infection through skin abrasions and mucous membranes introduce leptospires to conditions of the host venue, such as physiological osmolarity. Studies of the pathogen in culture have shown that the increase in osmolarity modulates gene expression, including the strong induction of the *lig* genes for the adhesins, LigA and LigB, which mediate adherence to the host via extracellular matrix proteins and the plasma protein, fibrinogen [9], [10], [11], [12], [13], [14]. Moreover, the binding of LigB to proteins involved in the repair of injured skin, such as fibroblast fibronectin and collagen type III, along with the inhibitory effect of LigB-fibrinogen binding on blood clotting [15], [16], suggest important roles for LigB early during infection. The successful entry into the circulation for dissemination to distal tissue also exposes the pathogen to the surveillance of host innate immunity, particularly the alternative pathway of complement activation early in an infection.

Spirochetes as well as other pathogens can actively counteract the bactericidal effects of the complement system by capturing key components and regulators, thereby disrupting the formation of functional complexes or accelerating the inactivation of others [17], [18], [19], [20], [21], [22], [23], [24]. Indeed, Johnson and colleagues described almost fifty years ago the resistance of pathogenic *Leptospira* strains to serum factors that killed nonvirulent strains [25], [26]. Subsequently, Cinco *et al.* found that the pathogenic strains acquired less of the terminal components of complement activation than avirulent leptospires [27]. Surface receptors have since been discovered that provide additional insight about the mechanisms used by pathogenic *L. interrogans* to evade innate immunity. Verma *et al.* showed that LfhA (now called LenA) binds factor H, the major inhibitor of the alternative pathway of complement activation [28]. LenA was also called Lsa24 and independently shown by Barbosa *et al.* to be a laminin-binding protein [29]; several proteins in microbial pathogens also bind extracellular matrix proteins or plasma proteins, such as fibrinogen and plasminogen, along with proteins in the complement system [28], [30], [31], [32], [33], [34]. Furthermore, Barbosa and colleagues found that LcpA in *L. interrogans* binds C4BP, the inhibitor of the classical and lectin pathways of complement activation [35]. Plasminogen binding has been shown for LenA also and other *L. interrogans* receptors, which could help to inhibit host immunity [36], [37], [38]. Another new study has found that the Lig proteins also interact with the immune system by binding the regulatory proteins, factor H and C4BP [39]. We now report that the expanded repertoire of the LigB adhesin in *L. interrogans* includes binding to the complement proteins, C3b and C4b, and show that LigB inhibits both the alternative and classical pathways and potentially helps the pathogen to counteract host innate immunity in concert with LenA and LcpA. Thus, LigB appears to exemplify a functional economy that has evolved in pathogenic leptospires to enhance virulence.

## Materials and Methods

### Assurance of ethical use of human samples and treatment of animals

This study was conducted according to the principles expressed in the Declaration of Helsinki, with informed written consent from participants and approval by the Institutional Review Board of the Research and Development Committee, Veterans Affairs Greater Los Angeles Healthcare System (PCC 2008-121778). In addition, this study was performed in strict accordance with the recommendations in the Guide for the Care and Use of Laboratory Animals of the National Institutes of Health, with Protocol Approval 0011-718 from the Institutional Animal Care and Use Committee of the Veterans Affairs Greater Los Angeles Healthcare System.

### Cells and proteins

The *ligB* transformant of *Leptospira biflexa* serovar Patoc strain Patoc I has been previously described [12], [15] and is maintained in serum-free Ellinghausen-McCullough-Johnson-Harris (EMJH) liquid medium under spectinomycin selection at 30°C. In the complement assays, fresh rabbit erythrocytes (Complement Technology, Tyler, TX) and antibody-sensitized sheep erythrocytes (Diamedix, Miami, FL) were used to measure activation of the alternative and classical pathways, respectively.

Recombinant His-tagged LigA and LigB proteins from *L. interrogans* serovar Copenhageni strain Fiocruz L1-130 have been previously described [11], [15]. The purified human complement and regulator proteins, C3b, C4b, C4BP, and factor H were obtained from Complement Technology.

### Sera and antibodies

Complement-active normal human sera were from Diamedix. Heat inactivation was done at 56°C for 30 min. Antisera from confirmed leptospirosis patients in acute and convalescent phases were obtained under guidelines for the protection of human subjects and were provided by Dr. Albert Ko, Yale University [3]. Rabbit polyclonal antibodies for Lig proteins were produced in-house following guidelines for the protection of research animals.

### Effect of LigB on viability of *L. biflexa* treated with serum complement

LigB expression by the transformed *L. biflexa* was verified by Western analysis as previously described [15]. In order to assess the viability of the transformed saprophytes, the conditions of serum concentration and time and temperature of treatment were adapted from previous studies that showed the susceptibility of untransformed *L. biflexa* to complement [25], [27], [40]. The *ligB* transformant and its parental strain transformed with the empty vector (2×10^7^ cells) were incubated separately in microcentrifuge tubes containing 100 µL serum-free EMJH with 0 to 20% (v/v) normal, non-immune human serum at 30°C for 5 min; heat-inactivated serum was tested also. Complement activation was stopped on ice for 1 min followed by the ten-fold dilution of a 20-µL aliquot with serum-free EMJH. The diluted cells were cultured at 30°C for four to six days, when both motile and non-motile leptospires were counted under darkfield microscopy. The results are presented as percentages of the live motile cells observed in the cultures treated with no serum (phosphate-buffered saline, PBS).

### ELISA of LigB binding to complement proteins

Recombinant His-tagged LigB proteins were assayed for binding to complement C3b and C4b. Each human complement protein, C3b or C4b (1 µg), was immobilized in microtiter wells by overnite incubation in 100 µL PBS, pH 7.2, at 4°C. Nonspecific binding sites were saturated with Protein-Free Blocking Buffer (Thermo Scientific, Rockford, IL). Up to 1 µM LigB in 100 µL 2% (w/v) bovine serum albumin-PBS, pH 7.2, was incubated at 37°C for 1 h. Following the removal of unbound LigB with three washes of PBS, complement-binding LigB was detected with a mouse monoclonal antibody for the His-tag (EMD Biosciences, San Diego, CA) at room temperature and measured by spectrophotometry at 450 nm of a 30-min conversion of tetramethyl benzidine (1-Step Turbo TMB, Thermo Scientific) by horseradish peroxidase conjugated to an antibody for mouse IgG (EMD Biosciences). Avidity was estimated with an apparent K_d_ derived as the concentration of LigB that gives half-maximal binding.

The effects of rabbit antibodies to Lig as well as antisera from confirmed leptospirosis patients on LigB-complement interaction were also determined. Antiserum was incubated at the indicated dilution with Lig protein in 2% bovine serum albumin-PBS, pH 7.2, for 1 h at 37°C prior to the complement-binding reaction above.

### Effect of LigB on innate immunity pathways

Two cellular assays were used to examine the effect of LigB on host immunity. The lysis of antibody-sensitized sheep erythrocytes by human serum complement was used to measure the classical pathway of activation. The procedure was adapted from the EZ Complement Cells CH50 kit (Diamedix) as previously described [32]. Concentrations of recombinant LigB were preincubated with 3 µL normal human serum or heat-inactivated serum in 20 µL PBS, pH 7.2, on ice for 1 h. The LigB and serum proteins were added to 3 mL of the sheep red blood cells and incubated at room temperature for 1 h. Following centrifugal sedimentation of the remaining intact cells, the released hemoglobin in 150 µL of the supernatant was measured at 415 nm in a microplate reader. The results are compared to hemolysis in the absence of LigB protein (100%). Serum inactivated at 56°C for 30 min produced negligible hemolysis. The alternative pathway was assayed by the lysis of fresh rabbit erythrocytes with human serum complement as previously described [32]. LigB protein was incubated with 7.5 µL normal human serum or heat-inactivated serum in 20 µL PBS, pH 7.2, on ice for 30 min prior to the addition of gelatin veronal buffer, 14 mM MgCl_2_, 14 mM EGTA (to suppress the classical pathway; data not shown), and the rabbit red blood cells (all from Complement Technology) in a final volume of 70 µL at 37°C for 30 min. The reaction was stopped with 1 mL cold veronal buffer, centrifuged, and the released hemoglobin in the supernatant measured in a microplate reader at 415 nm.

### Statistical analysis

The mean and its standard deviation or range from independent determinations (n, as indicated) are shown. Comparisons of means were made with parametric or non-parametric tests as indicated, where a two-tailed P<0.05 was considered to be statistically different.

## Results

### LigB inhibits complement-mediated killing of *L. biflexa*

Pathogenic *Leptospira* spp. are resistant to killing by normal mammalian serum, which in contrast readily disposes of avirulent strains and non-pathogenic species, such as the saprophyte, *L. biflexa*, through the activation of complement in the early defense system of host innate immunity [25], [26], [27], [40], [41], [42]. We tested the hypothesis that the LigB adhesin of virulent *L. interrogans* has also been adapted to mitigate innate immunity during the early stages of leptospirosis along with the other activities that have been proposed for it [12], [15], [16].

**Figure 1:**
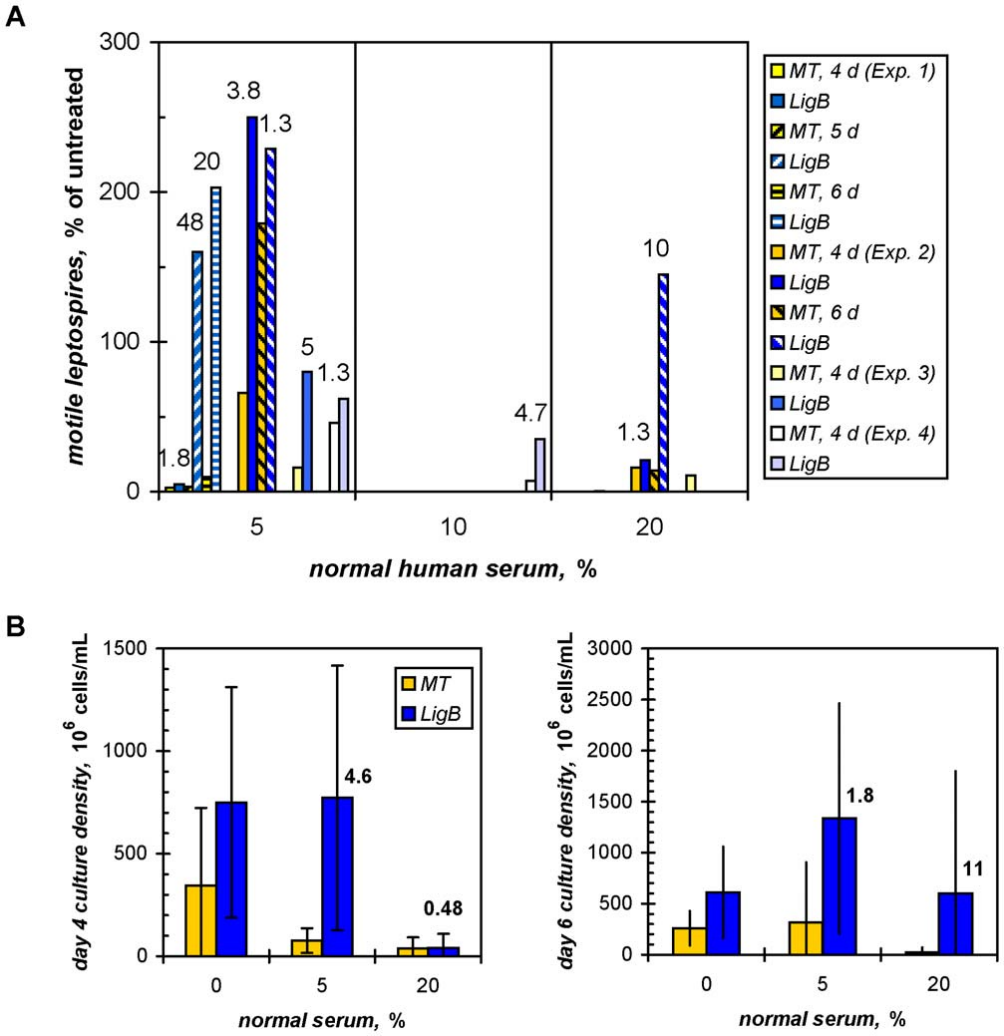
Ectopic LigB expression promotes survival of serum-treated *L. biflexa*. Viable saprophytes were counted following exposure to complement-active normal human serum as described in Materials and Methods. (**A**) The outgrowth of surviving *ligB*-transformed (*LigB*) *L. biflexa* was compared to that of cells transformed with the empty vector (*MT*) in four separate experiments (*Exp. 1* to *4*; each grouped and indicated by unique striping on *gold* background for the MT and *blue* for the LigB cells) four to six days following treatment with 5, 10, or 20% serum. Growth was calculated as the percentage of the untreated culture of the same strain, and the advantage gained by the LigB-expressing over the non-expressing cells is shown also as the fold increase in survival above each experimental pair. The cell numbers are presented in **Table S1**. (**B**) The average cell counts for the LigB-expressing and non-expressing cultures four and six days following treatment with 0, 5, or 20% serum are shown with the standard deviations (n = 4, day 4) or ranges (day 6). The fold difference of the expressing/non-expressing cell ratio after serum treatment over that for the untreated cultures is shown above the paired groups.

We used the highly complement-susceptible *L. biflexa* to examine the effect of LigB on the survival of leptospires following treatment with normal human serum. Whereas the normal saprophyte readily succumbed to a short incubation with serum, the singular addition of *ligB* from *L. interrogans* and its expression as a surface protein on *L. biflexa* [12] increased the survival of the transformant substantially (**Figure 1**). Four independent experiments are shown numbered *1* to *4*, comparing the survival of the empty-vector (*MT*) and LigB-expressing (*LigB*) transformants four to six days after treatment with 5, 10, and 20% serum (**Figure 1A** and **Table S1**). Although the results show the expected biological variability of the different preparations of cells and sera tested, they also demonstrate that the presence of LigB can partially rescue the cells from complement-mediated killing. The LigB-expressing cultures recovered 1.3- to 5-fold better than *L. biflexa* lacking LigB, reaching an average density of 7.7×10^8^±6.4×10^8^ leptospires/mL four days after treatment with 5% serum, which was comparable to the growth of the untreated LigB cells (7.5×10^8^±5.6×10^8^/mL; **Figure 1B**). The average culture density of the serum-treated non-LigB cells, 7.7×10^7^±6.0×10^7^/mL, was only 22% that of their unexposed counterparts (3.5×10^8^±3.8×10^8^/mL), which represented a 4.6-fold protective advantage overall for the LigB-expressing cells in the four experiments. Serum inactivated at 56°C for 30 min had no inhibitory effect on growth (data not shown). The time courses for the outgrowth of the two *L. biflexa* strains following their exposure to serum revealed greater protective effects of 48- and 20-fold for the LigB-expressing cells at 5 and 6 d, respectively, compared to 1.8-fold on day 4 in experiment 1. In experiment 2, the 3.8-fold advantage on day 4 leveled off to 1.3-fold two days later. There was an overall 1.8-fold protective effect with LigB 6 d after treatment with 5% serum in experiments 1 and 2 (**Figure 1B**).

The protective effect of LigB was less prominent with exposure to higher concentrations of serum (**Figure 1A** and **Table S1**). Although in some experiments, greater protection was associated with LigB expression, e.g., 4.7-fold 4 d after treatment with 10% serum and 10-fold 6 d after 20% serum treatment, the final culture densities were lower than with 5% serum treatment of the same cells. With one exception, the recovery of the serum-treated cultures was less than that of the untreated cells, reflecting the great complement sensitivity of the *L. biflexa* strains. **Figure 1B** summarizes the data by comparing the average growth of the LigB-expressing and non-expressing cells 4 and 6 d following exposure to 20% serum. As in the experiments with 5% serum, although statistical significance was not obtained, there was an 11-fold protective trend exhibited with LigB on day 6.

### LigB binds complement proteins, C3b and C4b

**Figure 2:**
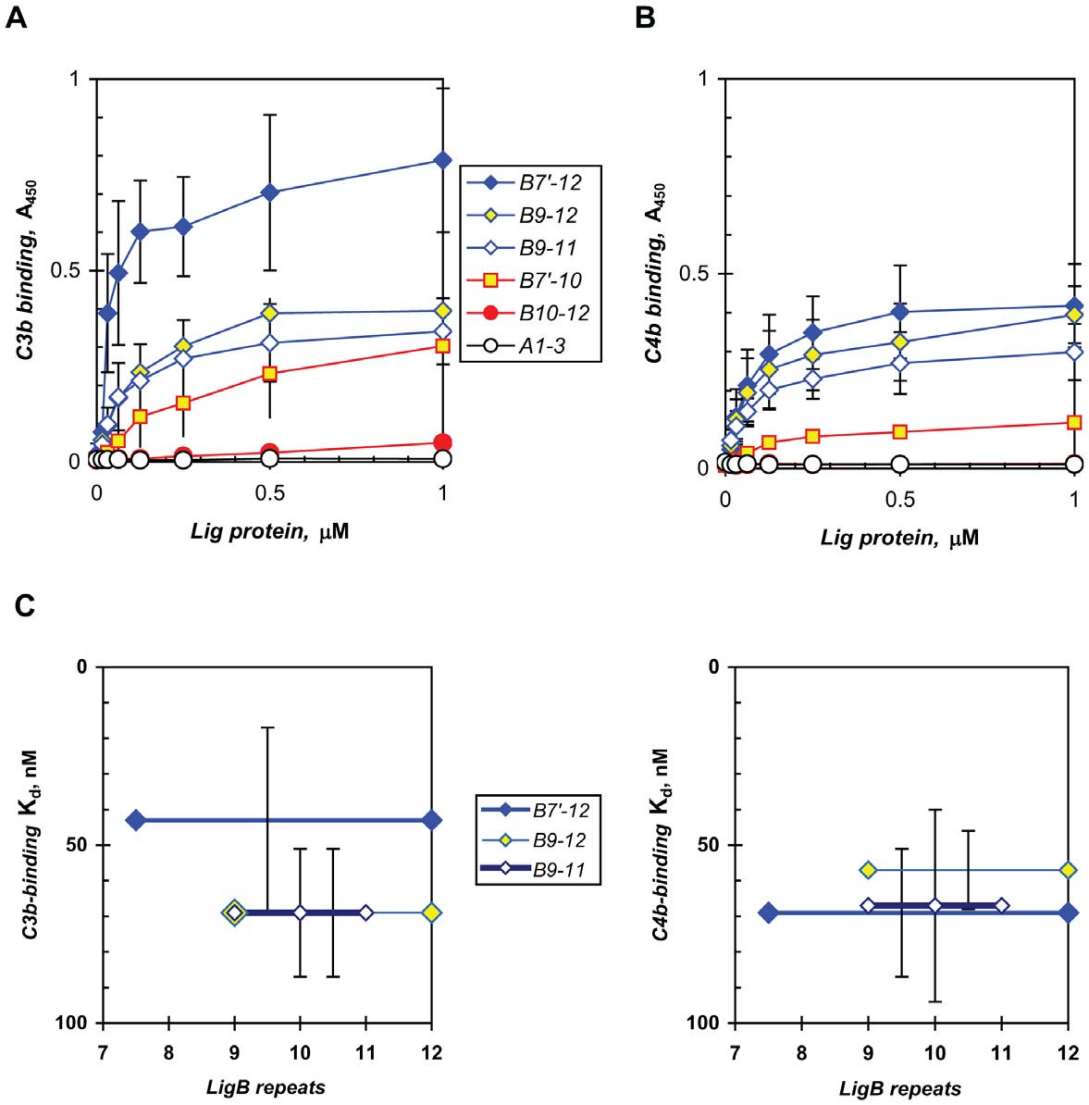
LigB binds complement proteins C3b and C4b. Recombinant Lig proteins were tested for interaction with human C3b (**A**) and C4b (**B**) in an ELISA described in Materials and Methods. The means and standard deviations or ranges (uncapped error bars) from multiple independent determinations (indicated in the text) comprising the cumulative binding curves are shown. Individual representative assays are shown for the inactive proteins, including LigB10-12, which is obscured by LigA1-3 in (**B**). The relative avidities of the binding proteins are discussed in the text, with the importance of LigB repeats 9 to 11 shown in (**C**), where the high-avidity LigB proteins are displayed on inverted scales of their mean apparent dissociation constants (standard deviations for n = 3 to 6). There is no statistically significant difference among these LigB proteins in their binding of each complement protein (ANOVA, P>0.05).

Given the mitigating effect on complement-mediated killing associated with the ectopic expression of LigB in *L. biflexa*, we examined the possibility of LigB interaction with C3b, a key component of complement in the antibody-independent alternative pathway of innate immunity. The recombinant LigB7′-12 protein that we have characterized as an adhesin in *L. interrogans* along with other functions that could mediate early host interactions in the transmission of leptospirosis also binds C3b with high avidity, exhibiting an apparent K_d_ of 43±26 nM (n = 6; **Figure 2A**). The half-repeat shorter LigB8-12 produced a similar binding curve (data not shown). In comparison with the other Lig proteins tested, the avidity of LigB9-11 and LigB9-12 is equivalent to that of LigB7′-12 (K_d_ of 69±18 nM, n = 3, P>0.05, Mann-Whitney test), thus mapping the major C3b-binding region within repeats 9 to 11. Weak- or non-binding Lig proteins are also shown, including LigB7′-10, LigB10-12, and LigA1-3, which contains the N-terminal three repeats in common between LigA and LigB. LigA7′-13, the LigB counterpart consisting of the LigA-unique repeats, binds C3b weakly (data not shown).

LigB also binds C4b, which is part of the antibody-dependent classical pathway (**Figure 2B**). This interaction is similar to that with C3b, giving a K_d_ of 69±18 nM (n = 4) for LigB7′-12. The binding region for C4b is also mapped within repeats 9 to 11, with K_d_s for LigB9-11 and LigB9-12 of 67±27 nM (n = 5) and 57±11 nM (n = 3), respectively, being indistinguishable from that for LigB7′-12 (P>0.05, non-parametric ANOVA). The weak-binding or inactive proteins, LigB7′-10, LigB10-12, and LigA1-3, are also shown.

**Figure 2C** is a comparative display based on the apparent dissociation constants of the major recombinant LigB proteins that are active in binding C3b and C4b. The statistical analysis of multiple independent measurements of the binding to C3b and C4b suggests the important domain(s) lie in repeats 9 to 11 of LigB.

### LigB inhibits the alternative and classical pathways of complement activation

**Figure 3:**
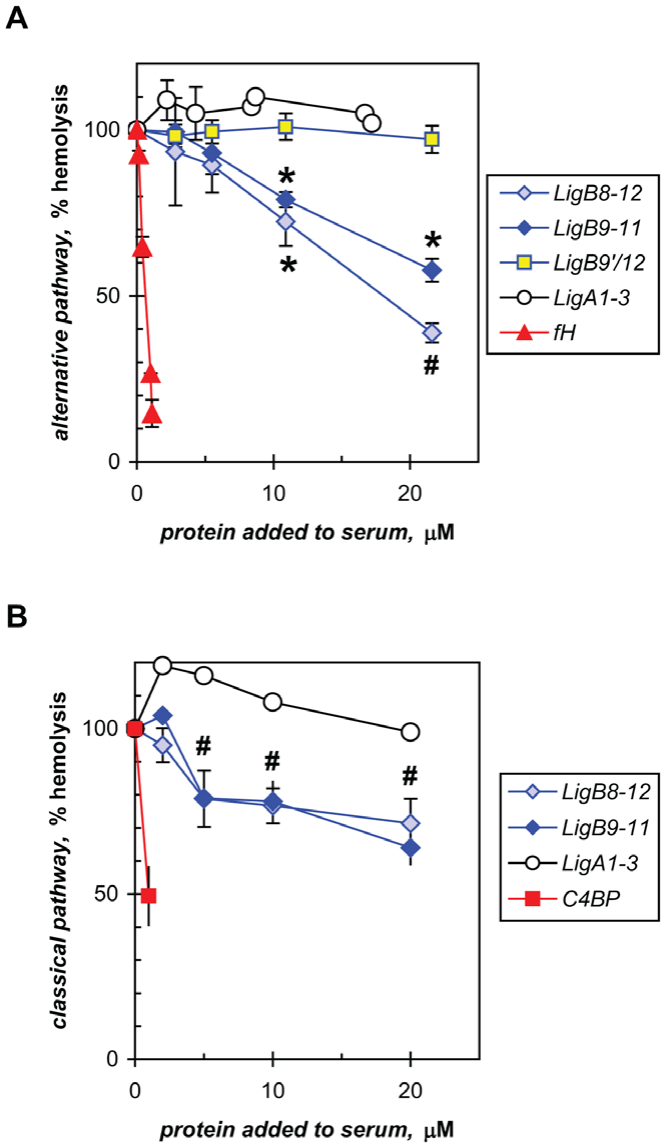
LigB inhibits complement activation. Recombinant Lig proteins were tested for effects on the complement-mediated hemolysis of erythrocytes with normal human serum as described in Materials and Methods. (**A**) The alternative pathway of activation was measured with rabbit red blood cells. C3b-binding LigB8-12 and LigB9-11 showed significant inhibition compared to non-binding LigB proteins grouped together as LigB9′/12 (see text for individual proteins; ANOVA (Kruskal-Wallis), P<0.05 (*), <0.01 (#)). Human factor H (*fH*), the C3b-binding inhibitor of the alternative pathway, and non-binding LigA1-3 were also tested. The means and standard deviations from three to six independent determinations are shown. (**B**) The classical pathway was measured with antibody-decorated sheep erythrocytes and C4b-binding LigB proteins. The contrasting effects of human C4BP (*C4BP*), the C4b-binding inhibitor of this pathway and non-binding LigA1-3 are also shown. The means and standard deviations (n = 3 to 4) or ranges (uncapped error bars) are shown, with ANOVA (Dunnett post-test) performed for LigB8-12 (#, P<0.01). One hundred percent hemolysis was obtained in the absence of Lig or regulator proteins.

The direct effect of LigB on complement activation was determined with a cellular assay of the alternative pathway as described in Materials and Methods. The preincubation of recombinant C3b-binding proteins, LigB8-12 and LigB9-11, with non-immune human serum inhibited complement-mediated hemolysis of the rabbit erythrocytes to 38.9±2.9% (n = 3) and 57.8±3.5% (n = 6), respectively, of complement activity in untreated serum (**Figure 3A**). LigB7′-12 was not produced in amounts sufficient for this assay. For reference, human factor H, a regulator of the alternative pathway that binds C3b, is shown to inhibit activation strongly at low concentrations, with 1.1 µM suppressing the level to 14.6±4.1% (n = 3) of untreated complement. Whereas heat-inactivated serum gave no lysis (data not shown), other recombinant LigB proteins (LigB9′-11, missing the N-terminal half of repeat 9, LigB10-12, LigB10-11, and LigB11-12) and LigA1-3 that do not bind complement or weakly so did not affect hemolysis (**Figure 3A** and data not shown). For statistical analysis, the data from the non-binding LigB proteins were combined to provide a composite negative control given the designation of LigB9′/12 identifying the range of LigB repeats tested.

A cellular assay of the classical pathway for the effect of LigB gave similar results (**Figure 3B**), with the recombinant C4b-binding proteins, LigB8-12 and LigB9-11, decreasing complement activation to 71.4±7.4% (n = 4) and 64% (range 9%), respectively, of untreated serum. A non-binding protein, such as LigA1-3, did not affect the complement-mediated hemolysis of antibody-sensitized sheep red blood cells. The classical pathway regulator that binds C4b, human C4BP, lowered activity to 49.4% (range 8.8%) with 1.0 µM.

### Inhibition of complement binding by patient antisera and rabbit antibodies

**Figure 4:**
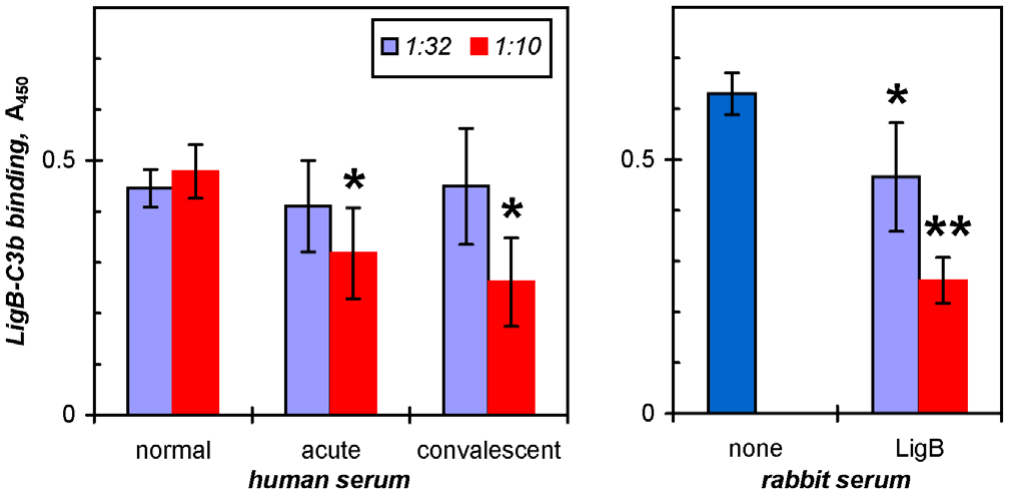
Leptospirosis patient and LigB-specific rabbit antibodies inhibit Lig-C3b binding. Pooled antisera from patients with *acute* infections of *L. interrogans* or *convalescent* patients or rabbit *LigB* antibodies were incubated at the indicated dilutions with 0.125 µM LigB7′-12 for 1 h prior to the ELISA for C3b binding. The means and standard deviations from three (human) to six (rabbit) determinations are shown. Statistically significant decreases from the level of C3b binding with normal human sera or in the absence of antibody are indicated where P<0.05 (_*_) and P<0.01 (_**_).

LigB is expressed by *L. interrogans* in a leptospirosis infection; the antisera from acute patients recognize LigB [43] and were thus tested along with rabbit antibodies to recombinant LigB for their effects on the LigB-C3b interaction characterized above (**Figure 4**). Pooled sera from either acute or convalescent patients inhibited C3b binding by LigB7′-12 as much as 33 and 46%, respectively, with the serum concentrations tested (P<0.05, ANOVA with Dunnett post-test). The rabbit polyclonal antibodies for LigB also interfered with C3b binding up to 60% (P<0.01).

## Discussion

Virulent transmission of a pathogen such as *L. interrogans* hangs in the balance of the dynamic interplay it engages in with the host, which along with the physical barriers of an integumentary system and mucosal membranes also presents a complex, hostile internal environment that includes innate immunity, the formidable system for surveillance and the destruction of intruders early in an infection. The microbe in turn does not arrive overmatched, with its own armament that includes the capability to directly subvert complement activation. Notable examples from human pathogens, such as Gram-positive *Staphylococcus* and *Streptococcus* as well as the spirochetes, *Borrelia* and *Leptospira*, are the surface receptors and secreted proteins that bind components and regulators of the host complement system [28], [30], [31], [32], [33], [34], [35], [36], [37], [44], [45], [46], [47], [48], [49], [50], [51], [52], [53], [54], [55], [56], [57], [58]. The binding of complement-cascade proteins can directly interfere with their assembly into functional complexes through displacement or conformational change or destabilize and prompt the decay of complexes. The capture of host regulatory proteins can provide the pathogen their respective modulatory and cofactor activities. In addition to acquiring complement components and regulators from the host, pathogens bind other host proteins, such as plasminogen, that lead to the degradation of important proteins of the immune system, such as C3 and IgG [21].

The ability to evade the immediate challenge from the alternative pathway of innate immunity is crucial for a successful infection. This is reflected in the resistance of pathogenic *Leptospira* in contrast to the great vulnerability of non-pathogenic *Leptospira* to the bactericidal effect of normal mammalian sera [25], [27], [40]. In this study, we tested the hypothesis that the functions of LigB in *L. interrogans* early in infection include mitigating innate immunity. A recent model of ectopic expression of the LigB adhesin on the surface of *L. biflexa* exhibited enhanced adhesion of the saprophyte to host extracellular matrix proteins [12], [15]. This model also provided a rigorous test for the effect of LigB on the viability of a cell that can be killed quickly even with 2% normal serum [25], [27]. Although significant biological variability was observed, the results showed overall that the expression of LigB inhibited the lethal effect of serum on the saprophyte (**Figure 1**). We further demonstrate that this cellular effect can be recapitulated at the molecular level with recombinant LigB proteins that bind C3b and C4b directly (**Figure 2**) and indeed inhibit both the alternative and classical pathways of complement activation in hemolytic assays with erythrocytes (**Figure 3**). Whereas *L. biflexa* is susceptible to the alternative pathway only [40] and Lig has been shown to bind factor H also [39], the protective effect provided by the ectopic expression of LigB appears to be due to the cell-surface binding of C3b and/or factor H.

The LigB interactions with C3b and C4b involve repeats 9 to 11, the region found to be important in fibronectin and fibrinogen binding [15], suggesting the use of an adaptable binding domain that serves multiple functions in the pathogenesis of leptospirosis. On the surface, this would seem to be inefficient, with competition among the multiple ligands of comparable binding affinities [11], [15]. However, an adaptable domain that is suitably surface-exposed could allow each LigB to bind the first ligand it recognizes during infection. Thus, with sufficient cell surface density of the domain, its multiple activities could help to counteract multiple, simultaneous homeostatic processes occurring at an infection site. Although LcpA in *L. interrogans* has only been reported to bind C4BP, factor H appears to share its binding site on LenA with laminin and plasminogen [36]. Laminin and plasminogen also have overlapping binding sites on Lsa20 in *L. interrogans* [57]. Pathogen-bound plasminogen could help to mitigate innate immunity, because its plasmin derivative cleaves C3b [23], [30], [38], [59]. Examples of other proteins in which complement regulators share a binding region with other host proteins include factor I- and fibrinogen-binding ClfA in *Staphylococcus aureus* [31], [60], plasminogen- and fibronectin-binding PfbB in *Streptococcus pneumoniae* [58], and C5a- and fibronectin-binding C5a peptidase in *Streptococcus agalactiae* [34].

Although LigB binds C3b and C4b with nanomolar avidity, the protection of *L. biflexa* from complement-mediated killing with LigB expression and the inhibition of erythrocyte lysis by recombinant LigB were modest. The level of ectopic expression of LigB by *L. biflexa* is comparable to the expression by *L. interrogans* [12], [15], which was confirmed in this study (data not shown). Thus, the partial protection of *L. biflexa* could reflect the roles of additional factors in the serum resistance of *L. interrogans*, such as LenA and LcpA.

The modest effect of recombinant LigB on complement activation in the hemolytic assays compared to those exhibited by factor H and C4BP suggests mechanistic differences. The previously characterized regulators also bind C3b and C4b, respectively, but they also serve as cofactors for factor I in cleaving C3b and C4b, leading to the destabilization of the C3 and C5 convertases that are necessary to finish all three complement pathways [20], [21], [22], [27], [30], [35], [40], [48], [51], [54], [61]. It is thus possible that LigB acts in concert with LenA, LcpA, and also LigA, all of which bind factor H or C4BP. Although LigA can occur as a slightly smaller N-terminal-truncated extracellular protein [9], it is unknown how the function of these leptospiral proteins may be affected by their relative abundance and membrane topography with respect to the cell surface sites that are prone to opsonization by C3b and C4b. Thus, the role of LigB may differ according to its proximity to C3b and C4b molecules on the leptospiral surface. In binding the two complement proteins, LigB could directly interrupt or destabilize convertase assembly by non-enzymatic means. LigB could also cooperate with neighboring LenA and LcpA and their capture of factor H or C4BP to enzymatically inactivate the complement pathways.

The recent discovery that the Lig proteins also bind factor H and C4BP [39] suggests the possibility that these serum proteins could compete with C3b and C4b for interaction with LigB under physiological conditions, and that part of the inhibition of complement activation observed in the hemolytic assays was due to the LigB proteins binding to factor H or C4BP. Given that the regulator-binding sites in LigB have not been defined, it remains to be determined whether factor H or C4BP binding played any part in the results observed here with the truncated LigB9-11 protein. However, whereas the N-terminal fragment of LigA and LigB binds factor H and C4BP [39], the N-terminal protein tested here, LigA1-3, did not affect complement activation in both the alternative and classical pathways. Moreover, whereas the apparent K_d_s for LigB-C3b and -C4b binding are in the low nanomolar range, these have not been determined for factor H and C4BP binding by Lig. Although Lig apparently is not a cofactor in the factor I-mediated processing of C3b and C4b as are factor H and C4BP, respectively [39], its binding to C3b and C4b could still hinder the assembly or stability of C3 and C5 convertases. It is certainly noteworthy that LigB interacts with both C3b and C4b along with both regulators, factor H and C4BP. The relative roles of LigA, LigB, LenA, and LcpA in immune evasion by *L. interrogans* remain to be determined.

LigB may be viewed as a leptospiral analog of a host-membrane complement control protein, such as complement receptor 1, which also binds C3b and C4b in disrupting the structure and function of the convertases involved in complement activation [62]. In lacking the cofactor activity for C3b and C4b cleavage, LigB differs from the host fluid-phase regulators, factor H and C4BP, which also bind C3b and C4b, respectively. However, by virtue of its additional ability to bind factor H and C4BP, LigB can mediate the acquisition of the cofactor activity for the pathogen as can LenA and LcpA. Thus, LigB and LigA, LenA, and LcpA could collectively help pathogenic leptospires to fill the self-protective role that is performed for the host by its cell-surface complement control proteins, complement receptor 1, membrane cofactor protein, and decay-accelerating factor, in the disruption of convertase assembly and the destabilization of convertases.

The LigB-C3b interaction is inhibited by patient antisera and Lig-specific rabbit antibodies (**Figure 4**). The presence of inhibitory antibodies in acute infections suggests that the complement-mitigating role of LigB would have to occur early in an infection before the antibodies are produced, indeed when the alternative pathway is an important part of host defense.

The multiple activities we have identified to date for LigB are consistent with functions that are important early in establishing a leptospirosis infection, such as attachment to the host and mitigation of host homeostasis and defense, including blood coagulation, platelet aggregation, and innate immunity. It appears then that pathogenic *Leptospira* spp. have adopted the functional economy of employing LigB as an adaptable interface to interact with multiple host proteins in the highly dynamic environment of an infection site.

## Supporting Information

Table S1 **Effect of LigB expression on serum treatment of** ***L. biflexa*** **.**
(PDF)
