# Supplementary material for: Multiple Activities of LigB Potentiate Virulence of Leptospira interrogans: Inhibition of Alternative and Classical Pathways of Complement
Source: PLoS One. 2012 Jul 23;7(7):e41566. doi: 10.1371/journal.pone.0041566 (PMC3402383; doi:10.1371/journal.pone.0041566)
Supplement: Table S1 — Effect of LigB expression on serum treatment of L. biflexa . (PDF) [file pone.0041566.s001.pdf]

Table S1. Effect of LigB expression on serum treatment of *L. biflexa*.

		Culture density, cells/mL[a]					
		**MT**[b]			**LigB**		
**NHS**[c]	**Experiment**	***4 d***	***5 d***	***6 d***	***4 d***	***5 d***	***6 d***
0	1	1.8×10^8	2.8×10^8	1.7×10^8	1.6×10^8	1.8×10^8	3.8×10^8
	2	1.0×10^8		3.4×10^8	5.6×10^8		8.3×10^8
	3	9.1×10^8			1.5×10^9		
	4	1.9×10^8			7.8×10^8		
5	1	4.8×10^6	7.2×10^6	1.7×10^7	7.8×10^6	3.2×10^8	7.7×10^8
	2	6.6×10^7		6.1×10^8	1.4×10^9		1.9×10^9
	3	1.5×10^8			1.2×10^9		
	4	8.7×10^7			4.8×10^8		
10	4	1.4×10^7			2.7×10^8		
20	1	0	0	6×10^5	0	0	0
	2	1.6×10^7		4.8×10^7	1.2×10^8		1.2×10^9
	3	1.0×10^8			0		

[a]Densities were determined as described in Materials and Methods, with those below the detection limit of 2×10^5 listed as 0.

[b]Abbreviations: *MT*, cells transformed with empty vector; *LigB*, *ligB* transformants; *NHS*, normal human serum.

[c]serum concentration in % (v/v).
